# Follow-up of individualised physical activity on prescription and individualised advice in patients with hip or knee osteoarthritis: A randomised controlled trial

**DOI:** 10.1177/02692155241234666

**Published:** 2024-02-26

**Authors:** Regina Bendrik, Lena V Kallings, Kristina Bröms, Margareta Emtner

**Affiliations:** 1 Department of Public Health and Caring Sciences, General Practice, 174463 Uppsala University, Uppsala, Sweden; 2 Centre for Research and Development, 214437 Uppsala University/ Region Gävleborg, Gävle, Sweden; 3 Department of Physical Activity and Health, 42750 the Swedish School of Sport and Health Sciences, GIH, Stockholm, Sweden; 4 Department of Medical Sciences, Respiratory, Allergy and Sleep Research, Uppsala University, Uppsala, Sweden

**Keywords:** Osteoarthritis, physical activity, physical function, behaviour change technique, accelerometer, physical activity on prescription

## Abstract

**Objective:**

Compare the long-term effects of two different individualised physical activity interventions in hip or knee osteoarthritis patients.

**Design:**

Randomised, assessor-blinded, controlled trial.

**Setting:**

Primary care.

**Subjects:**

Patients with clinically verified hip or knee osteoarthritis, <150 min/week with moderate or vigorous physical activity, aged 40–74.

**Intervention:**

The advice group (n = 69) received a 1-h information and goalsetting session for individualised physical activity. The prescription group (n = 72) received information, goalsetting, individualised written prescription, self-monitoring, and four follow-ups.

**Main measures:**

Physical activity, physical function, pain and quality of life at baseline, 6, 12 and 24 months.

**Results:**

There were only minor differences in outcomes between the two groups. For self-reported physical activity, the advice group had improved from a mean of 102 (95% CI 74–130) minutes/week at baseline to 214 (95% CI 183–245) minutes/week at 24 months, while the prescription group had improved from 130 (95% CI 103–157) to 176 (95% CI 145–207) minutes/week (p = 0.01 between groups). Number of steps/day decreased by −514 (95% CI −567–462) steps from baseline to 24 months in the advice group, and the decrease in the prescription group was −852 (95% CI −900–804) steps (p = 0.415 between groups). Pain (HOOS/KOOS) in the advice group had improved by 7.9 points (95% CI 7.5–8.2) and in the prescription group by 14.7 points (95% CI 14.3–15.1) from baseline to 24 months (p = 0.024 between groups).

**Conclusions:**

There is no evidence that individualised physical activity on prescription differs from individualised advice in improving long-term effects in patients with hip or knee osteoarthritis.

## Introduction

The level of physical activity is low in people with hip or knee osteoarthritis compared to the general population.^1^ It has been shown that if no treatment is offered, depression and worsening of radiographic osteoarthritis were correlated to a decline in physical activity (steps per day) 2 years later in individuals with or at risk of knee osteoarthritis and that those who were obese or had comorbidities walked significantly less at baseline.^2^ It is well known that physical activity interventions benefit osteoarthritis symptoms and general health. There is evidence of reduced pain, improved function and improved quality of life for 2 to 6 months after various physical activity interventions.^3,4^

According to systematic reviews with meta-analyses, the effects of physical activity interventions decline over time and sustained long-term effects are rare.^5,6^ However, comprehensive interventions individualised to the patient may increase long-term physical activity.^7,8^ In the Fit & Strong study,^7^ participants with improved physical activity at 12 and 18 months had received education combined with aerobic walking and resistance training for 22 sessions and a total of 36 h. All participants also developed individualised plans for long-term maintenance of physical activity.^7^ Another study by Pister et al. found that physical activity had improved after 15 months when the participants were offered 18 sessions with behaviour-graded activities, along with seven booster sessions over the following year.^8^

Other less comprehensive studies, which were individualised to the patient and included behaviour change techniques, had improved physical activity after 12 to 18 months.^9,10^ However, overall there are few published long-term studies based on behaviour change techniques in patients with lower limb osteoarthritis.^11^ In meta-analyses, if behaviour change techniques were used, beneficial long-term effects (≥12 months) have been found in adults with overweight and obesity^12^ and in adults at risk of chronic disease.^13^ International recommendations for patients with hip or knee osteoarthritis promote an individualised, patient-centred approach to improve physical activity.^14,15^ There is currently limited evidence of whether this intervention is beneficial in the long-term.^5,6^

The aim of this study was to compare the long-term effects (12 and 24 months from baseline) on physical activity level, physical function, pain and quality of life of two different, individualised, physical activity interventions in patients with osteoarthritis in the hip or knee.

## Method

In a parallel group with an assessor-blinded, randomised controlled trial, a 6-month intervention with physical activity on prescription (prescription group) was evaluated 6, 12 and 24 months after baseline compared to a 1-h advice session on physical activity (advice group). The study took place between June 2010 and December 2016. The Regional Ethical Review Board in Uppsala approved the study (DNR2010/001), which was conducted in accordance with the CONSORT guidelines for randomised controlled trials and registered at ClinicalTrial.gov (NCT02387034).

The sample size required was estimated in order to ensure sufficient statistical power for the analysis of the effects of physical activity measured as steps per day with an accelerometer. Based on a previous similar study,^16^ we assumed a mean difference of 600 steps per day between the prescription group and the advice group in the treatment effect, a standard within-group deviation of 1200 steps and a correlation of 0.75 between assessments in the same person before and after the interventions. A two-tailed t-test on the difference in effect between the two groups had the aim of achieving a desired power of 80% (p = 0.05) if the sample size was approximately 70 patients per group.

Randomisation was carried out by an assessor not involved in the study. Sealed and opaque envelopes in groups of 10, five for the prescription group and five for the advice group, were distributed to the health care centres. The physiotherapist who carried out the intervention opened the envelope to see the group to which the patient would belong. The physiotherapist was consequently not blinded to the group to which the patient was assigned. The assessor who measured the participants before allocation and at 6, 12 and 24 months was blinded for the allocation.

This study evaluated the patients at 12 and 24 months. Results from baseline to 6 months have been presented elsewhere,^17^ but will be presented in this study as comparison.

Patients with hip or knee osteoarthritis were recruited between 2010 and 2014 from a primary care in a town in central Sweden. Inclusion criteria were age, 40–74 years old, a verified clinical diagnosis of osteoarthritis in the hip or knee and a low level of self-reported physical activity (<150 min at moderate intensity or <75 min at vigorous intensity per week) at the time of screening.^18^ Exclusion criteria were hip fracture or a history of hip or knee replacement, meniscal injury, cruciate ligament injury, neuropathic pain in the leg, rheumatoid arthritis, severe cardiovascular disease or cancer and those who could not communicate in Swedish. Full details concerning the randomisation and the interventions have been described previously.^17^

The physiotherapists at the health care centres delivered the interventions. The contents of the two interventions are described in brief below:

Patients in the advice group received a 1-h session with comprehensive oral and printed information based on the book *Physical Activity in the Prevention and Treatment of Disease*,^19^ including the importance of daily activity for long-term health; and were also informed that physical activity is an effective way to cope with osteoarthritis and that physical activity is not associated with a higher risk of joint damage and flare ups. The individually tailored physical activity included advice to undertake aerobic activities (e.g. walking or cycling) three times per week for at least 30 min, as well as muscle-strengthening activities in daily life (e.g. to use stairs and focus on the legs when rising from a chair). Each patient could choose the preferred type of activities and the intensity of those activities.

The advice group was based on the behaviour change techniques *information about health consequences* and *goalsetting*.^20^ A discussion was held on potential harm and benefits associated with physical activity to enable the patient to make smart choices based on facts and not fears (*information about health consequences*). In addition, aerobic activities three times per week for 30 min and muscle-strengthening activities in daily life were the outcomes they were supposed to achieve (*goalsetting*).

Patients in the prescription group received the same 1-h of information and an additional, individualised, written physical activity prescription and subsequently four follow-up appointments for 6 months. The Swedish method of ‘Physical Activity on Prescription’ (S-PAP) was used.^21^ The prescription was based on what had been discussed and mutually decided on in the individual patient-centred dialogue. The written prescription spelled out the type, frequency and dose of physical activity and provided a form for self-monitoring of activities. Additional behaviour change techniques used in the prescription group were *action-planning, self-monitoring of behaviour, review of behaviour goals and graded tasks.*^20^ The prescription group was supported in formulating a *goal*, for example, play with my grandchildren on the floor, or be able to go up and down the stairs. Together with the physiotherapist, the patient discussed and decided how to act to achieve the goal and planned when, where and how the physical activities should be performed (*action-planning*). They were also instructed to self-monitor their behaviour by writing down their daily activities (*self-monitoring of behaviour*) until the next follow-up. At the individual follow-ups, the patient and physiotherapist evaluated the physical activity behaviour and new goals and activities were planned or adjusted (*review of behaviour goals, graded tasks*).

In addition, the patients in both groups also received *feedback* on the results of the performance-based tests of function at baseline, 6, 12 and 24 months. They became aware of their capacity when they saw how far they walked during the 6-min walk test, how many times they got up from the chair in the 30-s chair-stand test and whether one leg was weaker in the maximal step-up test.

**Figure 1.**
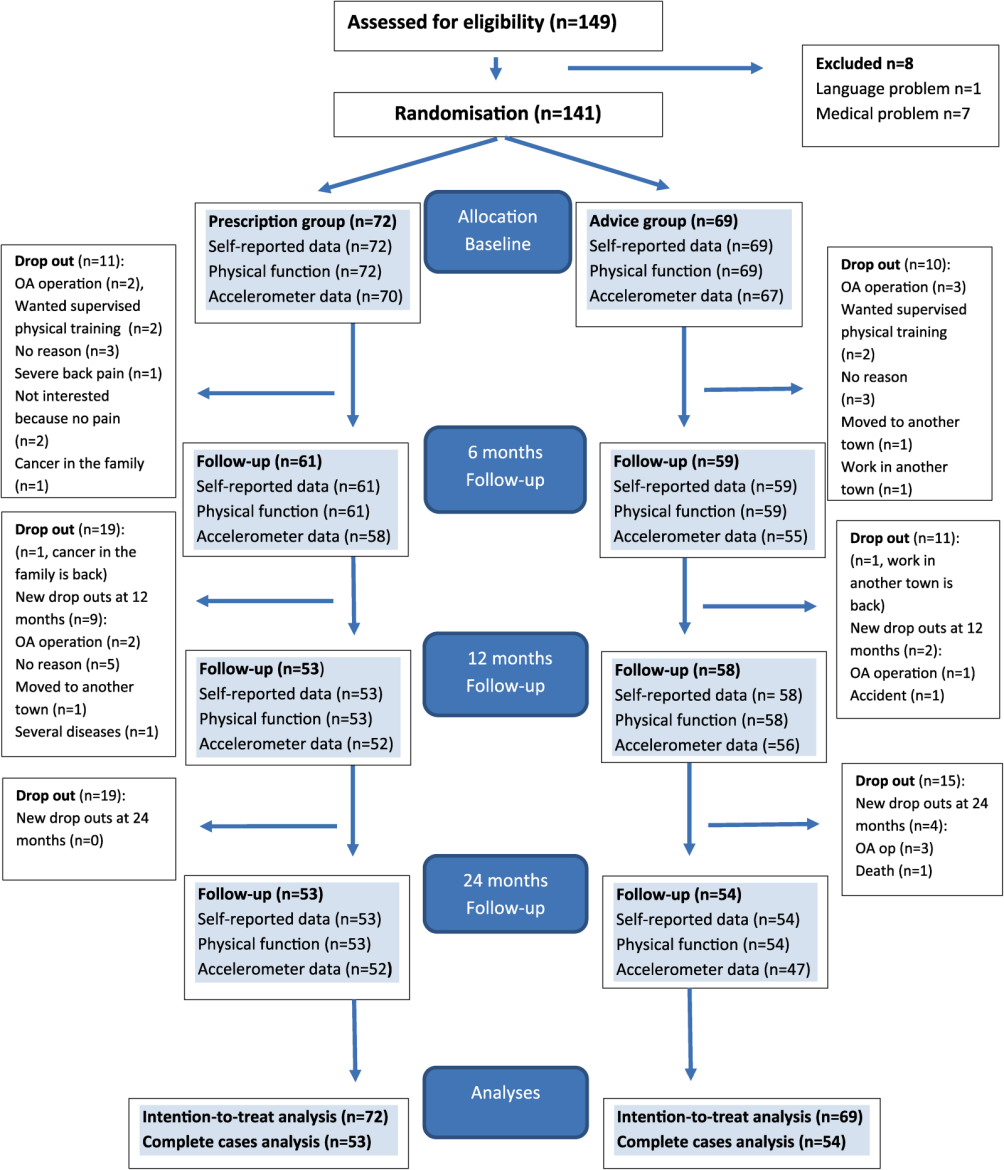
Enrolment, randomisation, dropout and follow-up at 6, 12 and 24 months.

Participant characteristics were assessed as part of the baseline questionnaire. Weight and height were measured and radiologic assessment of osteoarthritis was performed at baseline.^22^ Primary endpoint was 24 months, but outcomes were also assessed at 6 and 12 months (Figure 1). Primary outcomes were physical activity and secondary outcomes were physical function, pain and quality of life.

### Primary outcomes

- Self-reported physical activity: *Activity minutes* were assessed with two questions about exercise and everyday activity during a typical week. ‘How much time do you spend during a typical week on physical training that makes you feel short of breath, for instance, running, jogging or ball games?’ and ‘How much time do you spend during a typical week on daily physical activities such as walking, cycling or gardening?’ Both questions have five to seven categorical answer alternatives, and the answers were added up to produce total activity minutes. This was obtained by multiplying exercise by two to account for a proposed higher intensity (minutes in exercise × 2) and adding time in daily physical activity (minutes in everyday activity × 1).^23^ A clinically meaningful outcome could not be assessed, but the number of participants achieving at least 150 activity minutes per week was evaluated.^18^
- Accelerometer-assessed physical activity: *Steps per day* and *moderate and vigorous intensity physical activity (MVPA)* defined as  ≥ 3 metabolic equivalent of tasks (MET) were assessed with a three-axis accelerometer with health sensors, the SenseWear Armband Mini MF-SW (Body Media, Pittsburgh, Pennsylvania, USA). The SenseWear Armband software 9 was used.^24,25^ Full details of the SenseWear Armband have been described previously.^17^ Patients wore the sensor on the upper triceps 24 h a day for seven consecutive days. A valid day was counted as a day with 90% of 24 h as wear time. Accelerometer data were eligible if the patient had worn the sensor on at least four valid days. A clinically meaningful outcome could not be assessed.

### Secondary outcomes

- Physical function. *Six-min walk test* was used,^26^ and a clinically meaningful change of  ≥ 14.0 m was evaluated.^27^ A *30-s chair-stand test* was used,^26^ and a clinically meaningful change of  ≥ 2.0 repetitions was evaluated.^28^ A *maximal step-up test* was used.^29^ A clinically meaningful change could not be assessed. The number of participants achieving at least a step-up height of 24 cm was evaluated. It has been shown that less than 24 cm correlated to limitations in common daily activities such as climbing stairs, kneeling and walking more than 2000 m.^30^
- Pain and quality of life: Osteoarthritis-related pain and quality of life were assessed with the *Hip disability and Osteoarthritis Outcome Score (HOOS)*^31^ and *Knee injury and Osteoarthritis Outcome Score (KOOS)*,^32^ which is reported on a scale from 0 (worst) to 100 (best). A clinically meaningful change of ≥ 10 points was evaluated.^32^

### Statistical analyses

Baseline comparisons between groups are presented descriptively in Table 1. The outcomes were analysed with the intention to treat method and all participants including those with missing data and those who were not fully protocol compliant were included. To account for repeated measures, the following outcomes were analysed using linear mixed model^33^: activity minutes per week, steps per day, 6-min walk distance, repetitions in the 30-s chair-stand test, maximal step-up height, pain and quality of life. The model included time (baseline, 6, 12 and 24 months) and group (physical activity on prescription or advice) as categorical fixed factors, interactions between time and group, random intercepts and an unstructured covariance matrix. Missing data were largely due to participant attrition and appear to be either missing completely at random (not related to missing or observed data) or missing at random (not related to missing data). Marginal means were computed based on the estimated coefficients in the linear mixed models and presented with a 95% confidence interval (95% CI). Due to a high level (30%) of missing data in the accelerometer-assessed outcomes at 24 months, moderate and vigorous intensity physical activity in total minutes per day and moderate and vigorous intensity physical activity in 10-min bouts per day are presented separately, and steps per day were only included in the linear model for comparison. Missing data in moderate and vigorous intensity physical activity, total per day and in 10-min bouts per day, were imputed with the last case carried forward and analysed by Wilcoxon’s signed-rank test within groups (from baseline to 6, 12 and 24 months) and by the Mann–Whitney *U*-test of change between the groups. A two-sided P-value of less than 0.05 was set for statistical significance. Results are presented as between-group differences with mean and 95% confidence interval (95% CI) or median and quartiles. All analyses were performed with the use of Jamovi version 1.6 15.0 (IBM, New York, USA).

**Table 1.** Participant characteristics at baseline.

Characteristics	Randomised (n = 141)	
	Prescription group (n = 72)	Advice group (n = 69)
Women, n (%)	56 (78)	46 (67)
Age (years), mean (SD)	59.7 (8.6)	60.9 (7.9)
Body mass index (kg/m²), median (Q1, Q3)	31.0 (27.7, 33.5)	30.2 (26.8, 33.4)
Location osteoarthritis		
Hip, n (%)	19 (26)	18 (26)
Knee, n (%)	53 (74)	51 (74)
Radiographic OA severity, Kellgren–Lawrence score^a^, n (%), n = 137		
Score 0–2	58 (83)	55 (82)
Score 3–4	12 (17)	12 (18)
Comorbidity		
Depression, n (%)	6 (8)	5 (7)
Heart disease^b^, n (%)	11 (15)	12 (17)
Asthma/COPD, n (%)	5 (7)	7 (10)
Severe obesity (body mass index (kg/m²) > 35), n (%)	10 (14)	12 (17)
Severe pain (not due to knee or hip), n (%)	4 (6)	3 (4)
Diabetes mellitus, n (%)	3 (4)	4 (6)
Lifestyle self-reported		
Current smoker, n (%)	6 (9)	7 (10)
Alcohol^c^, risky consumption, n (%)	6 (8)	6 (9)
Eating habits^d^, unhealthy eating, n (%)	7 (10)	9 (13)
Education		
Elementary school, n (%)	22 (31)	23 (33)
High school, n (%)	34 (47)	33 (48)
College/university, n (%)	16 (22)	13 (19)
Not meeting recommendations for physical activity^e^, n (%)	52 (72)	55 (80)
Activity minutes per week^f^ self-reported, median (Q1, Q3)	104 (45, 206)	75 (45, 120)
Accelerometer-assessed physical activity (n = 70 respective n = 67)		
Steps, number/median (q1, q3)	7531 (5319,9749)	7041 (5175, 9111)
MVPA, total minutes per day, median (q1, q3)	71 (32, 117)	64 (42, 119)
MVPA, minutes per day in bouts of 10 min, median (q1, q3)	31 (12, 61)	30 (11, 71)
Six-min walk test, (m), mean (SD)	501 (78.1)	510 (72.6)
30-s chair-stand test, (n), mean (SD)	11.3 (4.0)	11.4 (3.0)
Maximal step-up test (affected leg), (cm), mean (SD)	22.1 (6.6)	23.7 (6.9)
HOOS^7^/KOOS^h^, mean (SD)		
Pain	52 (16.7)	55 (16.7)
Quality of life	39 (17.8)	39 (15.7)

a Scores on the Kellgren–Lawrence scale range from 1 to 4, higher scores indicate more severe disease.

b Heart disease: myocardial infarction, angina pectoris or heart failure.

c Alcohol, female risky consumption defined as  ≥ 9 standard glasses/week or ≥4 standard glasses on one occasion one or more times per month. For men defined as  ≥ 14 standard glasses/week or ≥5 standard glasses on one occasion one or more times per month. A standard glass corresponds to 33 cl of beer, 12–15 cl of wine or just under 4 cl of hard liquor.

d Eating habits, unhealthy eating habits defined from a questionnaire index as low consumption of fruits, vegetables and fish and high consumption of sweets, chips, buns and cakes and soft drinks.

e Self-reported <150 min/week of moderate physical activity or <75 min/week of vigorous physical activity.

f Activity minutes are (minutes in exercise × 2) + (minutes in every-day physical activity × 1).

g HOOS = Hip disability and Osteoarthritis Outcome Score, ranges from 0 (worst) to 100 (best).

h KOOS = Knee injury and Osteoarthritis Outcome Score, ranges from 0 (worst) to 100 (best).

## Results

A total of 141 patients were included (Figure 1); 111 patients (79%) were followed up at 12 months and 107 patients (76%) at 24 months. Baseline characteristics are presented in Table 1. Knee osteoarthritis was presented by 74% and mean age was 60.3 (8.3) in the patients included.

**Figure 2.**
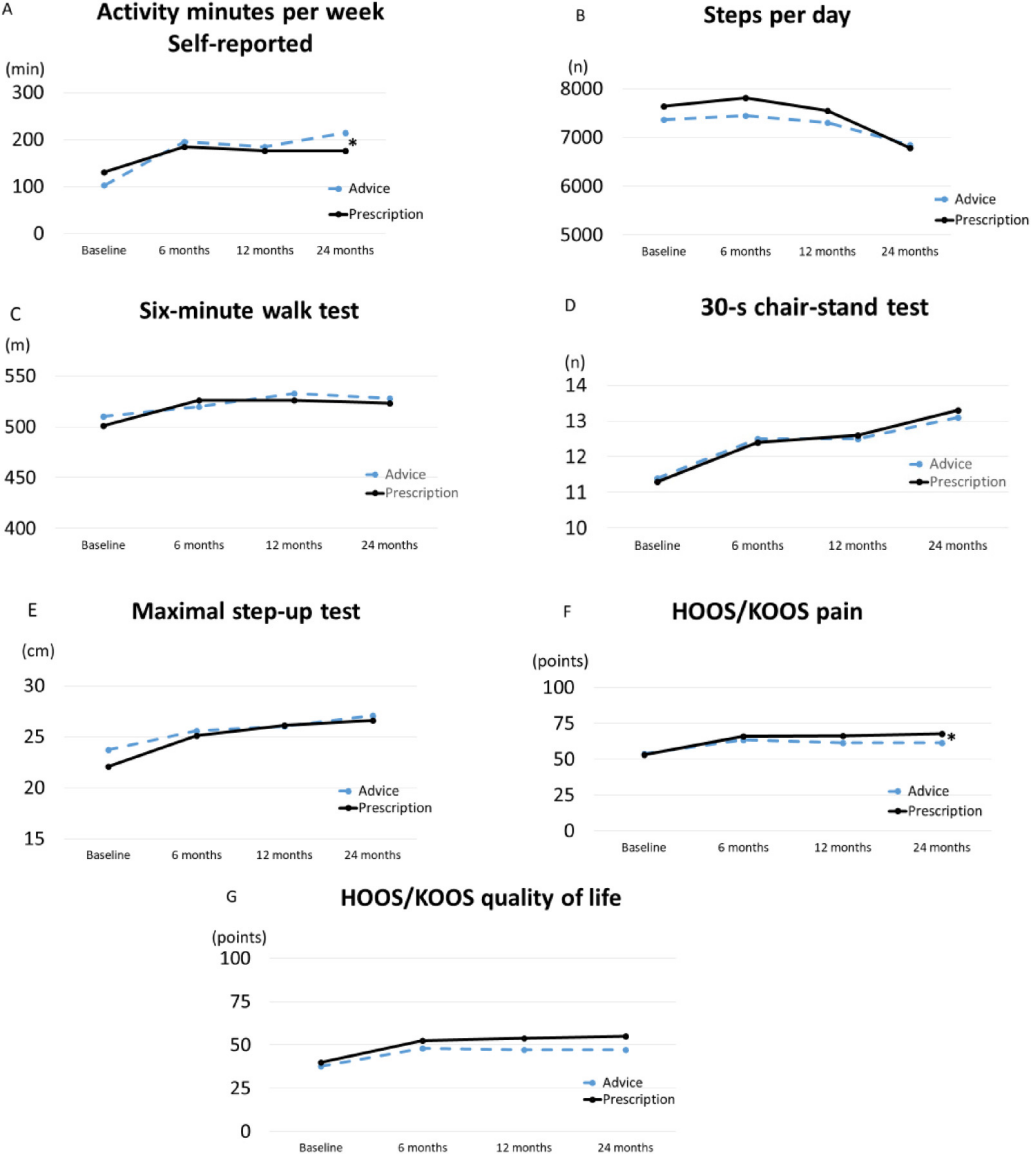
Estimated marginal means from a linear mixed effects model in patients with hip or knee osteoarthritis at baseline, 6, 12 and 24 months (n = 141 at baseline, n = 120 at 6 months, n = 111 at 12 months, n = 107 at 24 months). *p<0.05 between groups from baseline.

For self-reported physical activity, the advice group had a mean of 66 (95% CI 65–67) more activity minutes per week compared to the prescription group from baseline to 24 months (p = 0.01 between groups) (Table 2, Figure 2).

**Table 2.** Effects of prescription and advice interventions at baseline, 6, 12 and 24 months, and differences in change from baseline to 24 months, in physical activity (activity minutes per week and steps per day), physical function (6-min walk test, 30-s chair stand test and maximal step-up test), pain and quality of life. Estimated marginal means and 95% CI from a linear mixed model.

	Prescription group				Advice group				Mean difference	p-value^a^
	Baseline	6 months	12 months	24 months	Baseline	6 months	12 months	24 months	0–24 months^a^	
Activity minutes per week	130 (103–157) n = 72	185 (155–214)* n = 61	176 (145–207)* n = 53	176 (145–207)* n = 53	102 (74–130) n = 69	195 (165–225)* n = 59	185 (155–215)* n = 58	214 (183–245)* n = 54	66 (65–67)	0.010^b^
Steps per day	7636 (6895–8377) n = 70	7813 (7038–8589) n = 58	7543 (6755–8332) n = 52	6784 (5995–7573)* n = 52	7361 (6601–8121) n = 67	7443 (6657–8229) n = 55	7308 (6519–8096) n = 56	6847 (6034–659)* n = 47	−338 (−1148–473)	0.415
Six-min walk test (m)	501 (482–521) n = 72	526 (506–547)* n = 61	526 (505–547)* n = 53	523 (502–544)* n = 53	510 (490–530) n = 69	520 (500–541)* n = 59	533 (512–554)* n = 58	528 (507–549)* n = 54	−4 (−6—4)	0.665
30-s chair- stand test (n)	11.3 (10.6–12.2) n = 72	12.4 (11.6–13.3)* n = 61	12.6 (11.8–13.5)* n = 53	13.3 (12.4–14.1)* n = 53	11.4 (10.6–12.2) n = 69	12.5 (11.6–13.3)* n = 59	12.5 (11.8–13.5)* n = 58	13.1 (12.3–14.0)* n = 54	−0.3 (−0.6–1.2)	0.566
Maximal step-up test (cm)	22.1 (20.6–25.3) n = 72	25.1 (23.5–26.7* n = 61	26.1 (24.5–27.8)* n = 53	26.6 (25.0–28.2)* n = 53	23.7 (22.2–25.3) n = 69	25.6 (24.0–27.2)* n = 59	26.0 (24.4–27.7)* n = 58	27.1 (25.4–28.7)* n = 54	−1.1 (−1.2—0.5)	0.223
HOOS/KOOS pain (points)	53.0 (48.5–57.5) n = 72	66.0 (61.3–70.7)* n = 61	66.2 (61.3–71.1)* n = 53	67.7 (62.8–72.6)* n = 53	53.7 (49.2–58.3) n = 69	63.7 (58.9–68.5)* n = 59	61.5 (56.7–66.3)* n = 58	61.6 (56.7–66.5)* n = 54	−6.8 (−6.8–6.9)	0.024^c^
HOOS/KOOS quality of life (points)	40.0 (35.4–44.6) n = 72	52.5 (47.6–57.3*) n = 61	53.9 (48.8–58.9)* n = 53	54.9 (49.9–60.0)* n = 53	37.7 (33.0–42.4) n = 69	48.1 (43.2–53.1)* n = 59	47.2 (42.2–52.1)* n = 58	47.3 (42.2–52.3)* n = 54	−5.3 (−5.1—5.5)	0.098

a 24-month follow-up compared to baseline between-group difference (linear mixed model).

b In favour of the advice group.

c In favour of the prescription group, *=p* *< 0.05 within group from baseline.

Accelerometer-assessed physical activity measured as steps per day was about 7500 at baseline in both groups and stayed stable from baseline to 12 months, but decreased to about 6800 in both groups at 24 months (p = 0.415 between groups) (Table 2, Figure 2). Changes in moderate and vigorous intensity physical activity in total and in 10-min bouts per day showed no differences, neither between nor within groups from baseline to 24 months (p > 0.05) (Table 3).

**Table 3.** Accelerometer-assessed MVPA (≥3 METs), measured in total minutes and in bouts of 10 min per day in the prescription and advice groups, intention to treat, median (q1, q3).

	Prescription group n = 72	Advice group n = 69	Between-group difference, p-value
MVPA minutes per day, total minutes			
Baseline^a^	70 (29, 77)	62 (32, 70)	
6 months	69 (29, 78)	60 (31, 71)	0–6 m 0.821
12 months	72 (40, 121)	71 (42, 127)	0–12 m 0.920
24 months	67 (37, 99)	68 (44, 113)	0–24 m 0.517
MVPA minutes per day in 10-min bouts			
Baseline^a^	31 (22, 43)	31 (26, 37)	
6 months	35 (27, 36)	30 (30, 40)	0–6 m 0.253
12 months	30 (13, 62)	32 (10, 74)	0–12 m 0.333
24 months	25 (11, 51)	30 (11, 55)	0–24 m 0.390

MVPA: moderate and vigorous physical activity; MET: metabolic equivalent of tasks.

a p* *< 0.05 in group from baseline was not showed at any timepoints.

Results from the 6-min walk test, the 30-s chair-stand test, the maximal step-up test and the quality of life questionnaires displayed no between-group differences from baseline to 24 months (*p* > 0.05 between groups) (Table 2, Figure 2). For pain, the prescription group improved by a mean of 6.9 points (95% CI 6.8–6.9) more than the advice group, from baseline to 24 months (p = 0.024 between groups) (Table 2 and Figure 2).

At 24 months, 40 to 42% of the patients in both groups displayed a clinically meaningful improvement in physical function as measured by the 6-min walk test and the 30-s chair-stand test. About 30% in both groups displayed a clinically meaningful improvement in pain and quality of life (Table 4). In addition, at 24 months, the number of patients achieving self-reported physical activity at a level of 150 min per week of moderate or vigorous intensity (activity minutes) had increased from 20 to 54% in the advice group and from 28 to 43% in the prescription group (Table 4).

**Table 4.** Number of patients who reached healthy levels or a clinically meaningful improvement, n (%)^a^.

Variables	Prescription group n = 72	Advice group n = 69
Activity minutes per week, reached ≥150 min/week		
0 months	20 (28)	14 (20)
12 months	37 (51)	34 (49)
24 months	31 (43)	37 (54)
Maximal step-up test, reached ≥24 cm		
0 months	36 (50)	35 (51)
12 months	40 (56)	48 (70)
24 months	46 (64)	48 (70)
Six-min walk test, ≥ 14 m improvement		
0–6 months	34 (47)	29 (42)
0–12 months	36 (50)	34 (49)
0–24 months	29 (40)	28 (41)
30-s chair-stand test, ≥ 2 numbers improvement		
0–6 months	24 (33)	24 (35)
0–12 months	23 (32)	24 (35)
0–24 months	30 (42)	28 (41)
HOOS^b^/KOOS^c^ pain, ≥ 10 points improvement		
0–6 months	34 (47)	25 (36)
0–12 months	25 (35)	17 (25)
0–24 months	25 (35)	20 (29)
HOOS^b^/KOOS^c^ quality of life, ≥ 10 points improvement		
0–6 months	29 (40)	30 (43)
0–12 months	29 (40)	25 (36)
0–24 months	24 (33)	21 (30)

a If participant was missing at 6, 12 or 24 months, the result was scored as having not improved at all.

b HOOS = Hip disability and Osteoarthritis Outcome Score, ranges from 0 (worst) to 100 (best).

c KOOS = Knee injury and Osteoarthritis Outcome Score, ranges from 0 (worst) to 100 (best).

## Discussion

The findings show that there are only minor differences between the two physical activity interventions, individualised physical activity on prescription and individualised advice, when comparing the physical activity level and clinical outcomes at 12 and 24 months. Clinically meaningful improvements in physical function, pain and quality of life were displayed in 29 to 42% of the participants at 24 months.

Both groups improved self-reported physical activity from baseline to 24 months, whereas there were small decreases in accelerometer-assessed physical activity. The results are consistent with a meta-analysis evaluating 57 studies with at least 100 participants, which showed a low correlation between accelerometer-assessed and self-reported physical activity in healthy adults and individuals with diseases.^34^ This study is also in accordance with a meta-analysis evaluating individuals with osteoarthritis, which showed none or only a small increase in accelerometer-assessed physical activity after physical activity interventions.^35,36^ In a recent study evaluating individuals with knee osteoarthritis, the intervention group increased self-reported physical activity by 84 more minutes per week compared to the control group, whereas in accelerometer-assessed physical activity, none of the groups had improved, and there were no between-group differences.^10^

One important reason why the results differ between self-reported and accelerometer-assessed physical activities is that the accelerometer assesses absolute physical activity, while the self-reported activity depicts a relative intensity. Another reason is that the accelerometer has limitations in detecting all types of physical activity, for example, strength training and cycling.^34^ Our patients may have improved small incidental activities in daily life, such as standing up from a chair and walking up the stairs, which were probably not captured by the accelerometer. As self-reported questionnaires and accelerometers measure different aspects of physical activity, they complement each other and both are valuable.^34^ In addition to measures of physical activity, performance-based tests of function should also be used in the evaluation of patients with hip or knee osteoarthritis.^26^

We found only minor differences in outcomes between the two interventions, which may depend partly on a comparison between two very similar interventions. The similarities were an individualised intervention based on the behaviour change techniques *information about health consequences*, *goalsetting* and *feedback*. In addition, the patients from both groups could choose their own physical activities. Although the physical activity on prescription intervention was more extensive, including four follow-ups and use of multiple behaviour change techniques, we did not find any increased physical activity in this group. The fact that our 1-h patient-centred counselling with a physiotherapist might be enough to increase physical activity in the long-term needs further investigation, as previous research has shown that extensive efforts are needed.^7,8,10^ We believe that considering patients’ preferences for physical activity and involving the patient in treatment decisions are core components of individualised patient-centred counselling.^37^ In addition, the *information about health consequences* behavioural change technique should be combined with a physical activity intervention.^38^ *Feedback* was mainly obtained from the results of the performance-based tests of function, which resemble daily activities and are easy to understand, and probably motivated the patients to continue with their chosen activities.^26,29^ *Feedback* was in a meta-analysis reported to be the most important behaviour change technique for maintaining long-term physical activity (≥12 months) in adults.^13^ *Goalsetting* has also been shown to be effective in increasing long-term physical activity (≥12 months).^11,12^

The strengths of the study were the patient-centred individualised physical activity interventions based on behaviour change techniques, physical activities that could be performed in daily life, the performance-based tests of function and the long-term follow-up of patients from a regular primary care setting. One important limitation was the lack of a proper control group. A control group with no intervention for 24 months would have been unethical with these patients. We should perhaps only have used the performance-based tests of function at baseline and 24 months so as to reduce the effect of feedback. Another limitation was the attrition rate and low adherence to the accelerometers. A type-2 error might have emerged as only 70% of the participants in accelerometer-assessed outcomes at 24 months were followed up. Although one inclusion criteria was <150 min of moderate or vigorous physical activity per week, the patients were quite physically active at baseline, with little room for major improvements. We believe that the results of this study are generalisable to current clinical practice even though the study was conducted some years ago. This approach may be beneficial in other settings, but the results would have to be confirmed by further studies.

Future research should use a proper control group to evaluate whether individualised physical activity interventions including behavioural change techniques can increase physical activity in the long term. However, the regression to the mean effect must be taken into account as it was shown that this was at least 10% when pain was assessed in individuals with knee osteoarthritis.^39^

In conclusion, this study demonstrated that individualised physical activity on prescription including four follow-ups does not offer long-term benefits in increasing physical activity over and above individualised advice given once.

**Clinical messages**

- There is still lack of evidence for any effective physical activity interventions that increase physical activity in osteoarthritis patients in the long term.
- Individual counselling with support to choose preferred physical activities that are easy to perform in daily life may be a beneficial approach for long-term maintenance.
